# Population genetic structure, introgression, and hybridization in the genus *Rhizophora* along the Brazilian coast

**DOI:** 10.1002/ece3.3900

**Published:** 2018-02-25

**Authors:** Patrícia M. Francisco, Gustavo M. Mori, Fábio M. Alves, Evandro V. Tambarussi, Anete P. de Souza

**Affiliations:** ^1^ Center for Molecular Biology and Genetic Engineering University of Campinas Campinas São Paulo Brazil; ^2^ São Paulo Agency for Agribusiness Technology Piracicaba São Paulo Brazil; ^3^ Institute of Biosciences São Paulo State University (Unesp) São Vicente São Paulo Brazil; ^4^ Department of Plant Biology Institute of Biology University of Campinas Campinas São Paulo Brazil; ^5^ Department of Forestry Engineering Midwestern State University Irati Paraná Brazil

**Keywords:** gene flow, hybrid zone, introgressive hybridization, mangrove, Rhizophoraceae

## Abstract

Mangrove plants comprise plants with similar ecological features that have enabled them to adapt to life between the sea and the land. Within a geographic region, different mangrove species share not only similar adaptations but also similar genetic structure patterns. Along the eastern coast of South America, there is a subdivision between the populations north and south of the continent's northeastern extremity. Here, we aimed to test for this north‐south genetic structure in *Rhizophora mangle,* a dominant mangrove plant in the Western Hemisphere. Additionally, we aimed to study the relationships between *R. mangle, R. racemosa,* and *R*. ×* harrisonii* and to test for evidence of hybridization and introgression. Our results confirmed the north‐south genetic structure pattern in *R. mangle* and revealed a less abrupt genetic break in the northern population than those observed in *Avicennia* species, another dominant and widespread mangrove genus in the Western Hemisphere. These results are consistent with the role of oceanic currents influencing sea‐dispersed plants and differences between *Avicennia* and *Rhizophora* propagules in longevity and establishment time. We also observed that introgression and hybridization are relevant biological processes in the northeastern coast of South America and that they are likely asymmetric toward *R. mangle,* suggesting that adaptation might be a process maintaining this hybrid zone.

## 1 INTRODUCTION

Mangrove species are plants that grow in the intertidal zone along rivers, estuaries, and shores between latitudes of approximately 30°N and 30°S (Hamilton & Casey, 2016). These trees and shrubs comprise a polyphyletic group of taxa that have evolved similar morphological, ecological, and physiological traits. These traits have enabled these species to inhabit anoxic and saline environments that are influenced by the tidal regime in tropical and subtropical areas. Examples of such convergent traits are viviparity, mechanisms that increase salt tolerance, specialized pneumatophore roots, and seawater‐based seed or fruit (propagule) dispersal (Ball, 1988; Duke, Ball, & Ellison, 1998; Tomlinson, 1986). Additionally, at the molecular phenotype level and, more specifically, at the transcriptome level, there are exceptional similarities in the gene expression profiles of mangrove species. These similarities suggest the occurrence of parallel evolution in two distantly related mangrove lineages to cope with a common environment (Dassanayake, Haas, Bohnert, & Cheeseman, 2009).

The resemblances among distantly related mangrove species are not limited to adaptations to an environment that is influenced by both land and sea. Different species also share similar genetic structure patterns at a wide range of geographic scales in many mangrove forest regions. Such similarities are apparent in the Indo‐West Pacific biogeographic region (IWP), an area rich in mangrove species in the Eastern Hemisphere (Duke, Lo, & Sun, 2002; Duke et al., 1998). In South‐East Asia, there is a clear divergence between populations from the western and eastern coast of the Malay Peninsula observed for *Ceriops tagal* (Perr.) C.B. Rob. (Rhizophoraceae) (Huang et al., 2012), *C. decandra* (Griff.) W. Theob. (Tan et al., 2005), *Bruguiera gymnorrhiza* (L.) Lamk. (Rhizophoraceae; Minobe et al., 2010), *Excoecaria agallocha* L. (Euphorbiaceae; Zhang et al., 2008), *Lumnitzera racemosa* Willd. (Combretaceae; Li et al., 2016), *Rhizophora apiculata* Blume (Rhizophoraceae; Yahya et al., 2014; Yan, Duke, & Sun, 2016), and *R. mucronata* Lam. (Wee et al., 2014). Although this pattern is not universal—for instance, it was not detected in other *Rhizophora* (Yan et al., 2016) and *Sonneratia* (Yang et al., 2016) species—it indicates that common extrinsic factors, such as superficial ocean currents (Wee et al., 2014) and sea‐level fluctuations (Yan et al., 2016), have shaped the organization and distribution of genetic diversity.

In the Atlantic East Pacific region (AEP), a biogeographic region with lower mangrove species diversity than the IWP, a similar phenomenon was observed at a comparable geographic scale. Along the eastern coast of South America, there is a pattern of genetic subdivision between populations north and south of the northeastern extremity of the South America continent (NEESA). This divergence pattern is shared by the sea‐dispersed plants *Rhizophora mangle* L. (Pil et al., 2011; Takayama, Tamura, Tateishi, Webb, & Kajita, 2013), *Avicennia germinans* L., *A. schaueriana* Stapf and Leechman ex Moldenke (Acanthaceae; Mori, Zucchi, & Souza, 2015) and *Hibiscus pernambucensis* Arruda (Malvaceae; Takayama, Tateishi, Murata, & Kajita, 2008). As in the IWP, the regional genetic structure in the AEP can be explained by superficial marine currents and responses to Pleistocene climate variations (Mori, Zucchi, Sampaio, & Souza, 2015; Mori, Zucchi, & Souza, 2015; Pil et al., 2011; Takayama et al., 2008). Despite the genetic structure shared by distantly related species, restricted geographic sampling and variation among different molecular marker sets can potentially influence the description of intraspecific genetic structure.

Here, we tested for the north‐south genetic structure pattern in this biogeographic region using *R. mangle,* a widespread, dominant mangrove species in the AEP biogeographic region, as the biological system. To assess the repeatability of previous results (Pil et al., 2011; Takayama et al., 2013), we developed a new set of microsatellite markers of the same class used previously to describe this genetic structure in this system. Additionally, we sampled plants from four and seven mangrove forest regions north and south of the NEESA, respectively, to improve the representation of the northern “population.” Finally, because AEP *Rhizophora* species exhibit semipermeable species boundaries (Cerón‐Souza et al., 2010, 2014; Takayama et al., 2013), it is possible that ancient and ongoing interspecific hybridization is a relevant evolutionary process in the northern population (Pil et al., 2011). As the existence of such a process could influence the observed patterns of genetic structure, we also tested for the presence of a hybrid zone. In some areas to the littoral north of the NEESA, the three AEP *Rhizophora* species *R. mangle, R. racemosa* Meyer, and *R. *×* harrisonii* Leechman (*pro sp*.) are sympatric, although the distributions of the last two species are disjunct (Menezes, Berger, & Mehlig, 2008). Additionally, to the best of our knowledge, *R. racemosa* and *R. *× *harrisonii* have not been recorded south to the NEESA.

## 2 MATERIAL AND METHODS

### 2.1 Plant material and sampling strategy

We distinguished the species in the field primarily based on reproductive morphological traits to minimize misidentification issues. At one extreme, *R. mangle* inflorescences exhibit 2–5 flowers each and no more than two orders of bifurcation. At the other extreme, *R. racemosa* inflorescences present up to 128 flowers each, with up to seven orders of bifurcation (Cerón‐Souza et al., 2010; Tomlinson, 1986). Furthermore, the latter species displays rounded flower buds, whereas the former shows pointed ones. *R. *× *harrisonii* identification was based on its three to five ordered branched inflorescences with up to 32 flowers and its apically pointed flower buds. This species has a morphology intermediate between those of *R. racemosa* and *R. mangle* (Tomlinson, 1986)*,* which is likely the outcome of ancient and ongoing gene flow between *R. racemosa* and *R. mangle* morphotypes (Cerón‐Souza et al., 2010; Takayama et al., 2013).

**Figure 1:**
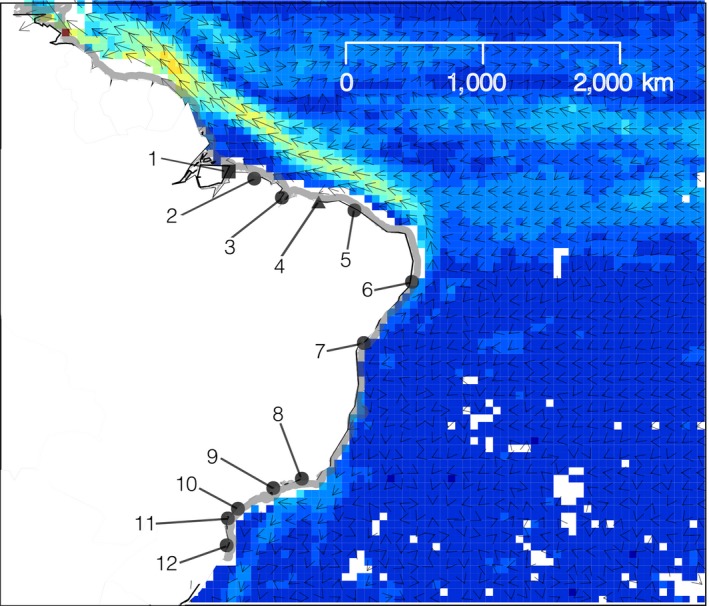
Geographic distribution of *Rhizophora* samples along the northwestern South American coast. Acronyms indicate the locations where plants morphologically identified as *R. mangle, R. *×* harrisonii,* and *R. racemosa* were sampled, according to Table 1. Circles, squares, and triangles represent sampling sites where only *R. mangle* was collected, where only *R. racemosa* and *R. *× *harrisonii* were sampled, and where the three species were obtained from, respectively. The average speeds of marine currents based on The Global Drifter Program (US National Oceanic and Atmospheric Administration—Atlantic Oceanographic & Meteorological Laboratory) are represented using arrows, whose slope indicates current direction, while the magnitude indicates current velocity. Additionally, we represent current velocity with colors, with higher temperatures indicating higher velocities for each cell

We collected visually healthy leaf samples from 318 specimens of *R. mangle* from 11 sites and 33 samples of *R. racemosa* and 37 samples of *R. *×* harrisonii* from two localities along the Brazilian coast, covering more than 4,900 km of coastline (Figure 1, Table 1). Sampling expeditions were conducted from June 2008 to December 2010. We recorded latitude and longitude using a global positioning system (GPS) receiver (Garmin 76CSx, WGS‐84 standard, Garmin International Inc., Olathe, KS, USA). Licenses (17159 and 17130) to collect the leaves of these species were obtained from the Instituto Brasileiro do Meio Ambiente e dos Recursos Naturais Renováveis (IBAMA, currently Instituto Chico Mendes de Conservação da Biodiversidade, ICMBio). Geographic information regarding the sampled species is provided in Table 1, where Rm, Rr, and Rh indicate *R. mangle, R. racemosa,* and *R. *×* harrisonii*, respectively, and the three‐letter codes indicate the localities from which the samples were obtained.

**Table 1:** Description of *Rhizophora* sampling locations along the northeastern South American coast

Number ID	Acronym	Locality (City, State)	Geographic Coordinates	Rm	Rr	Rh
1	MRJ	Soure, Pará	0° 43′ 26″ S, 48° 29′ 24″ W		RrMRJ (11)	RhMRJ (7)
2	PAR	Bragança, Pará	0° 49′ 12″ S, 46° 36′ 56″ W	RmPAR (30)		
3	ALC	Alcântara, Maranhão	2° 24′ 37″ S, 44° 24′ 22″ W	RmALC(31)		
4	PNB	Parnaíba, Piauí	2° 46′ 42″ S, 41° 49′ 20″ W	RmPNB (13)	RrPNB (22)	RhPNB (30)
5	PRC	Paracuru, Ceará	3° 24′ 47″ S, 39° 3′ 23″ W	RmPRC (34)		
6	TMD	Tamandaré, Pernambuco	8° 31′ 35″ S, 35° 0′ 48″ W	RmTMD (20)		
7	VER	Vera Cruz, Bahia	12° 59′ 1″ S, 38° 41′ 5″ W	RmVER (25)		
8	GPM	Guapimirim, Rio de Janeiro	22° 42′ 5″ S, 43° 0′ 26″ W	RmGPM (25)		
9	UBA	Ubatuba, São Paulo	23° 29′ 22″ S, 45° 9′ 52″ W	RmUBA (32)		
10	CNN	Cananéia, São Paulo	25° 1′ 12″ S, 47° 55′ 5″ W	RmCNN (35)		
11	PPR	Pontal do Paraná, Paraná	25° 34′ 30″ S, 48° 21′ 9″ W	RmPPR (24)		
12	FLN	Florianópolis, Santa Catarina	27° 34′ 37″ S, 48° 31′ 8″ W	RmFLN (49)		

The sampled populations are composed of individuals morphologically identified as *R. mangle* (Rm), *R. racemosa* (Rr), and *R. *× *harrisonii* (Rh) in the field, following previous studies describing this species complex (Cerón‐Souza et al., 2010; Tomlinson, 1986). The sample sizes are shown within parentheses. Sampling site acronym, municipality and state in Brazil and geographic coordinates are indicated for each site.

We deposited voucher specimens from every location in the University of Campinas (UEC) and Embrapa Amazônia Oriental (IAN) herbaria, which are both in Brazil. For genetic analyses, we sampled leaves from flowering trees that were at least 20 m from any other sampled trees and maintained the leaves in resealable zipper plastic bags containing silica gel. The leaf material was lyophilized and stored at −20°C prior to DNA isolation.

### 2.2 Molecular biology procedures

To identify and characterize new genetic markers, we developed a microsatellite‐enriched genomic library for *R. mangle* and *R. racemosa*. Using the DNeasy^®^ Plant Mini Kit (Qiagen, Hilden, DE, Germany) according to the manufacturer's instructions, we isolated the genomic DNA from one individual of each species sampled in northern Brazil (0°43′26″S, 48°29′24″W for *R. mangle* and 0°49′12″S, 46°36′56″W for *R. racemosa*). We constructed microsatellite‐enriched libraries for both species following a magnetic bead‐based method (Billotte, Lagoda, Risterucci, & Baurens, 1999) that our research group has used for other mangrove species (Mori, Zucchi, Sampaio, & Souza, 2010; Mori, Zucchi, & Souza, 2015). We designed 44 and 37 primer pairs based on microsatellite‐enriched libraries developed for *R. mangle* and *R. racemosa*, respectively, from which we obtained eight and three markers that were polymorphic (both within and among species), respectively (see Results). Additionally, we included one microsatellite marker that was previously developed for *R. mangle* (Rosero‐Galindo, Gaitan‐Solis, Cárdenas‐Henao, Tohme, & Toro‐Perea, 2002) and was consistently amplified across our sampled populations. Markers that presented intra‐ or interspecific polymorphism were employed in subsequent experiments, leading to different microsatellite marker sets (Table 2). For the entire sample, composed of *R. mangle, R. harrisonii,* and *R. racemosa* individuals, we used seven microsatellites, whereas for the dataset composed of only *R. mangle* or by *R. racemosa* and *R. harrisonii* individuals, we used two marker sets with eight microsatellites each (Table 2). For the entire sample, composed of *R. mangle, R. *× *harrisonii,* and *R. racemosa* individuals, we used seven microsatellites, whereas for the dataset composed of only *R. mangle* or by *R. racemosa* and *R. *× *harrisonii* individuals, we used two marker sets with eight microsatellites each (Table 2). We tested whether the number of loci used in each dataset was sufficient to discriminate between unique individuals using the *genotype_curve* function with 10,000 loci resampling in the package POPPR 2.3 (Kamvar, Javier, & Niklaus, 2014).

**Table 2:** Microsatellites used in this study

Marker name	All species	Rm	Rr & Rh	Size (bp)	Repeat motif	Primer sequence (5′–3′)	GenBank
M41^a^	P	P	P	210	(GA)_25_	F: TGGAAGGATTGTGGTAATTGGG	AF484998
						R: CATGTGGGTGTGCTCTGGG	
Rma3‐5	P	P	P	170	(TC)_18_	F: CAAGGTCAATGGGTGTAG	KJ740656
						R: AATGAAGCAAATAAGAGATAAG	
Rma3‐14	P	P	P	160	(TC)_18_	F: AAATGCATAAAAGTTGAAGATA	KJ740663
						R: AAAAGGATGTGATGAGACTGTT	
Rma3‐17	–	P	–	210	(TG)_15_	F: TTCATCACCAGCACCAAAGT	KJ740669
						R: TGACCTCGCAATCTACACAAA	
Rma3‐23	P	P	P	230	(CT)_19_	F: AAGTGGGTCATGTTTAGAA	KJ740675
						R: CTTATGGTATGTGTATTAGGTC	
Rma3‐37	P	P	P	240	(AG)_5_	F: AGGCCATTTATACTCTCACACC	KJ740686
						R: TTACGGCGAACCACACTT	
Rma3‐38	–	P	–	260	(AG)_5_…(AGA)_10_	F: TGGCAGATGTGTCTTCCTGA	KJ740686
						R: CCTCAGACTTGAATCAGCAGTG	
Rra1‐18	P	P	P	239	(TG)_5_(GT)_12_	F: TGTGGGTGCATGGATTAGATTTAT	KM870545
						R: CACGCGCCTTGGATTCATTT	
Rra1‐33	P	–	P	214	(AG)_11_	F: GACCAGTGAGTAAAAAGGGAGTAG	KM870558
						R: CTGGGCCATGCAATAGTGA	
Rra1‐35	–	–	P	355	(CA)_19_	F: TTCTGAGCTCAAATGTCT	KM870556
						R: TTCAGCCTCTTCCAATA	

Characteristics of the microsatellite markers employed for the *Rhizophora* species complex in southeastern South America are shown. Rm, Rr and Rh denote individuals morphologically identified in the field as *R. mangle*,* R. racemosa* and *R. *× *harrisonii*, respectively, following previous studies describing this species complex (Cerón‐Souza et al., 2010; Tomlinson, 1986). Rma3 and Rra1 indicate microsatellite markers developed for *R. mangle* and *R. racemosa,* respectively. P denotes polymorphic markers, whereas “–” indicates no amplification. The expected sizes based on the clone fragment, repeat motifs, primer sequences, and GenBank accession numbers are shown.

a The M41 marker was obtained from a previously developed microsatellite set (Rosero‐Galindo et al., 2002).

To amplify the fragments, we performed polymerase chain reactions of 20 μl containing 2 ng template DNA, 2 mM MgCl_2_, 50 mM KCl, 20 mM Tris‐HCl (pH 8.4), 0.2 mM dNTPs, 0.19 mg/ml bovine serum albumin (BSA), 0.15 mM primer and 1 U *Taq* DNA polymerase. PCR was performed according to a touchdown thermocycling program: 94°C for 2 min; 2× [10 cycles of 94°C for 1 min, 65°C (−1°C/cycle) for 1 min and 72°C for 2 min]; 18 cycles of 94°C for 1 min, 55°C for 1 min and 72°C for 2 min; and 72°C for 5 min. The amplified samples were genotyped by vertical electrophoresis using 6% denaturing polyacrylamide gels, and the bands were visualized using silver nitrate (Creste, Neto, & Figueira, 2001). The sizes of the resulting fragments were estimated by comparison with a 10 bp DNA ladder (Thermo‐Invitrogen, Carlsbad, CA, USA).

### 2.3 Hybrid detection and characterization

To maximize the number of evaluated loci, we considered different microsatellite sets for each analysis considering their amplification and polymorphism within or among species. For each dataset, we used ARLEQUIN 3.5 (Excoffier & Lischer, 2010) to test for linkage disequilibrium (LD) for all pairs of markers in each sample, with 10,000 permutations. Microsatellite analyses may present technical artifacts that bias population genetic parameter estimates; therefore, we used Micro‐checker 2.2.3 (Van Oosterhout, Hutchinson, Wills, & Shipley, 2004) with a confidence interval of 99% to detect the presence of null alleles, large allele dropout, and stuttering. As we did not observe consistent evidence of LD between microsatellites, null alleles, large allele dropout, or stuttering within samples across the samples, we carried out the subsequent analyses considering the entire dataset.

Due to the complex hybridization and introgression processes observed among *Rhizophora* species (Cerón‐Souza et al., 2010, 2014; Takayama et al., 2013), we performed genetic structure and hybrid identification analyses using a dataset that combined *R. mangle, R. *× *harrisonii,* and *R. racemosa* microsatellite genotypes prior to further intraspecific analyses. The choice of this approach was based on the “unified concept” that species are independently evolving metapopulation lineages (De Queiroz, 2007); as such, individuals of a given species are expected to be more related to each other than to individuals of a different species regardless of their geographic origin.

We used the Bayesian method implemented in STRUCTURE 2.3.4, assuming a model with correlated allele frequencies and admixture (Falush, Stephens, & Pritchard, 2003; Pritchard, Stephens, & Donnelly, 2000). For each number of groups (*K*) considered, which ranged from 1 to 10, we performed 30 independent Markov Chain Monte Carlo (MCMC) runs with 500,000 iterations, following a burn‐in period of 100,000 steps. We determined the *K* value that best explained our data at the uppermost hierarchical level, considering the log likelihood of each *K* (lnL) (Pritchard et al., 2000) and the ad hoc statistic Δ*Κ* (Evanno, Regnaut, & Goudet, 2005). Postprocessing of this model‐based population structure method was performed with CLUMPAK (Kopelman, Mayzel, Jakobsson, Rosenberg, & Mayrose, 2015). To complement this model‐based clustering method, we performed discriminant analysis of principal components (DAPC; Jombart, Devillard, & Balloux, 2010), a multivariate method implemented in the ADEGENET 2.0 R package (Jombart & Ahmed, 2011) that employs sequential K‐means and model selection to identify and describe genetic clusters. For this analysis, we considered *K* values from 1 to 50 and used the Bayesian information criterion (BIC) to identify meaningful *K*s that summarized our data. Additionally, we employed the *optim.a.score* function to balance discrimination power and overfitting.

To identify and classify eventual two‐generation hybrids as F_1_s, F_2_s or backcrosses, we used a different model‐based method implemented in NewHybrids 1.1 (Anderson & Thompson, 2002). We performed 10 independent MCMC runs with 500,000 steps following a burn‐in period of 100,000 iterations for different prior distribution combinations. We performed DAPC, STRUCTURE, and NEWHYBRIDS analyses considering all samples and those from the north coast of Brazil, because of the observed genetic structure pattern at its uppermost hierarchical level (see Results). Additionally, to evaluate the patterns of introgression between the clusters or hybrid classes inferred by these methods, we employed multinomial regression to estimate individual hybrid indices, corresponding to individual clines in genotype frequency for each marker along a genomic admixture gradient (Gompert & Buerkle, 2009). This regression was implemented using the INTROGRESS R package (Gompert & Alex Buerkle, 2010). As we obtained similar, but partially contrasting results between the STRUCTURE analysis and the DAPC and NEWHYBRIDS analyses (see Results), we estimated the hybrid indices for individuals collected from regions of sympatry that were identified as hybrids using at least one of these methods. As nonadmixed individuals, we considered *R. mangle* with STRUCTURE‐estimated ancestry values (*Q*‐values) higher than 0.80 for the typical *R. mangle* inferred cluster for the most likely scenario, *K = *2 (see Results) and individuals identified as *R. racemosa* in the field (that were assigned to the alternative cluster for the *K = *2 scenario). Microsatellite alleles were combined into classes of alleles with frequency differentials between the nonadmixed groups (i.e., species) equal to those obtained when each allele was considered separately. This approach reduces data complexity without distorting the genetic similarity between species or losing information (Gompert & Buerkle, 2009). We then compared the INTROGRESS results based on the observed data with those for simulated hybrids (F1s, F2s or backcrosses) between the parental species generated by the *hybridize* function of ADEGENET 2.0 (Jombart & Ahmed, 2011).

Finally, due to the complex pattern of hybridization and introgression among these *Rhizophora* species, we used Arlequin 3.5 (Excoffier & Lischer, 2010) to conduct hierarchical analysis of molecular variance (AMOVA) (Excoffier, Smouse, & Quattro, 1992). We tested competing a priori hypotheses based on the morphological identification of individuals and mangrove genetic structure patterns in the Neotropics (Mori, Zucchi, Sampaio, et al., 2015; Mori, Zucchi, & Souza 2015; Pil et al., 2011; Takayama et al., 2013). Additionally, we tested the a posteriori hypothesis that *R. mangle* is a separate group and that *R. racemosa* and *R. *×* harrisonii* constitute a different group (see Results) as well as the hypothesis of an additional subdivision within *R. mangle* (Table 3). As criteria for identifying the scenario that was best supported by our data, we employed the maximum variance among groups (Φ_CT_) and a significant departure from a random distribution obtained after 10,000 permutations.

**Table 3:** Analysis of molecular variance (AMOVA) for the a priori and a posteriori hypotheses regarding the *Rhizophora* species complex

Model	Hypothesized groupings	Φ_ST_	Φ_SC_	Φ_CT_	% Among groups
Model A	[Rm] [Rh] [Rr]	0.79135*	0.37759*	0.66477*	66.48
Model B	[Rm] [Rh + Rr]	0.79824*	0.37958*	0.6748*	67.48
Model C	[RmN] [RmS] [Rh + Rr]	0.70865*	0.23762*	0.61784*	61.78
Model D	[RmN − RmPRC] [RmS + RmPRC] [Rh + Rr]	0.72995*	0.21485*	0.65605*	65.61

Rm, Rr, and Rh denote individuals morphologically identified as *R. mangle*,* R. racemosa* and *R. *×* harrisonii*, respectively, in the field, following previous studies describing this species complex (Cerón‐Souza et al., 2010; Tomlinson, 1986). RmN and RmS denote the groupings of populations sampled north and south of the northeastern extremity of South America. RmPRC indicates samples of *R. mangle* from Paracuru, Ceará, Brazil, as in Table 1 and Figure 1. *p < 0.05.

### 2.4 Intragroup genetic structure

After describing the genetic structure at the interspecific level, we performed intraspecific genetic structure analyses of each inferred group (see Results). As we used a different dataset for each inferred group to maximize the genetic information evaluated, for each group, we investigated the presence of LD and microsatellite technical artifacts (stuttering, large allele dropout and null alleles) using the previously described methods (Excoffier & Lischer, 2010; Van Oosterhout et al., 2004). Again, we did not observe consistent evidence of LD nor technical artifacts across the sampled populations; therefore, we used the entire datasets for the subsequent analyses.

We estimated population summary statistics, including observed heterozygosity (*H*_O_), unbiased expected heterozygosity (*H*_E_), the number of private alleles (A), and the fixation index (*f*) using GenAlex 6.502 (Peakall & Smouse, 2012). We then tested for Hardy–Weinberg equilibrium with GENEPOP 4.2 (Rousset, 2008) with 5,000 dememorization steps in 500 batches with 5,000 iterations each.

To describe how the observed genetic variation was organized, we applied DAPC, using BIC to identify the *K* value that best explained our data and the *optim.a.score* function to avoid overfitting (Jombart et al., 2010). Additionally, we applied the clustering method implemented in STRUCTURE 2.3.3, assuming a model with correlated allele frequencies and admixture (Falush et al., 2003; Pritchard et al., 2000) and considering *K* values from 1 to 10. Thirty independent MCMC runs with 500,000 iterations and a burn‐in period of 100,000 was performed. The *K* value that most efficiently summarized our data was determined based on lnL (Pritchard et al., 2000) and Δ*Κ* (Evanno et al., 2005), and the STRUCTURE results were postprocessed using CLUMPAK (Kopelman et al., 2015).

For the *R. mangle* group, we also applied spatial principal component analysis (sPCA; Jombart, Devillard, Dufour, & Pontier, 2008), implemented in ADEGENET 2.0 (Jombart & Ahmed, 2011), to study the genetic variability in its spatial context. sPCA detects spatial genetic structure patterns that simultaneously present spatial autocorrelation and high variation without assuming Hardy–Weinberg equilibrium or linkage equilibrium among loci (Jombart et al., 2008). It also allows for identifying “positive” or “negative” autocorrelation, which respectively emerge from genetic similarity or spatial gradients that result from barriers to gene flow, isolation by distance or adaptation (“global structure”) and greater genetic differences among neighbors than expected (“local structure”) (Jombart et al., 2008). To test for global and local structure, we performed 10,000 permutations. We determined the connection network using the minimum distance that retained all sampling localities in this network.

## 3 RESULTS

### 3.1 New microsatellite isolation and characterization

We constructed microsatellite‐enriched genomic libraries composed of 96 positive clones for both *R. mangle* and *R. racemosa*. After sequencing each colony via automatic sequencing, we designed 81 primer pairs (44 based on the *R. mangle* microsatellite‐enriched library and 37 based on the *R. racemosa* library), which yielded six and three new polymorphic microsatellites considering intra‐ or interspecific levels for *R. mangle* and *R. racemosa,* respectively (Table 1). We discarded the remaining markers due to nonspecific banding during testing, unexpected amplification product sizes or nonamplification. The datasets we used presented sufficient power to discriminate between unique individuals (Figure S1).

### 3.2 Hybrid detection and description

**Figure 2:**
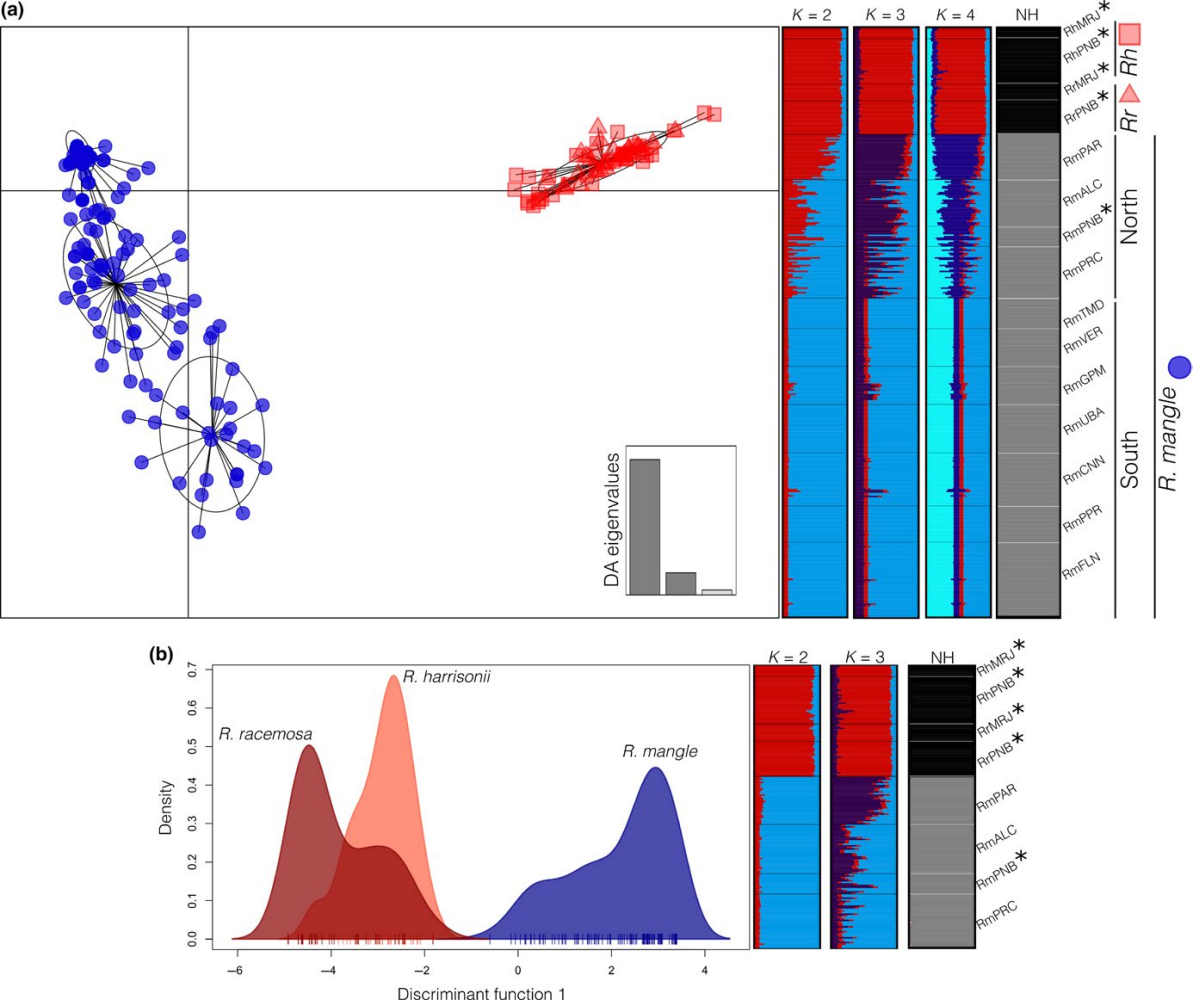
Hybridization and historic introgression in the Rhizophora species complex considering the entire sample (a) and only individuals from the northern coast of Brazil (b). From left to right: Scatterplot of the discriminant analysis of principal components (DAPC), where individuals morphologically identified as *R. mangle*,* R. × harrisonii*, and *R. racemosa* are represented as blue circles, red squares, and red triangles, respectively (a), or by colors (b). Bar plots showing STRUCTURE 2.3.4 Bayesian group assignments, in which each bar represents a single individual and colors represent proportional membership coefficients, for two (*K* = 2), three (*K* = 3) and four (*K* = 4) groups, considering the whole sample, or for *K* = 2 and *K* = 3, considering only the northern samples. Bar plots depict the NEWHYBRIDS 1.1 Bayesian hybrid class assignment, in which bars represent individuals; black represents one “pure species,” and gray represents the alternative “pure species (NH).”

Using seven microsatellite markers (Table 1), we genotyped 388 individuals from 12 localities, covering more than 4,900 km of coastline along the Atlantic coast of South America. The Bayesian clustering method implemented in STRUCTURE indicated two evolutionary scenarios, according to Δ*Κ*, that best explained our data: *K *=* *2 and *K *=* *4; however, the lnL approach indicated increasing lnL values from *K = *1 to *K *=* *4 (Figure S2). Regardless of the considered scenario with *K* ranging from 2 to 4 (Figure S2), the admixture proportions were concordant in separating *R. mangle* from *R. racemosa* and *R. *×* harrisonii* (Figure 2). When *K = *2, the Bayesian admixture proportions indicated that a substantial amount of *R. mangle* individuals from the zone of sympatry with *R. racemosa* and *R. *×* harrisonii* were admixed at a wide range of levels. However, assuming *K *=* *3 or *K = *4, the *R. racemosa* and *R. *× *harrisonii* individuals were drawn from a single gene pool, whereas the *R. mangle* trees were represented by different groups, suggesting the existence of intraspecific genetic structure (Figure 2).

The multivariate clustering method DAPC also supported *K *=* *4 as a fair summary of the data we obtained. Moreover, it showed that *R. mangle* is composed of three gene pools that are each distantly related to individuals of *R. racemosa* and *R. *× *harrisonii*, which comprise a single, more homogeneous genetic cluster (Figure 2). Hybrid classification based on NEWHYBRIDS also indicated that *R. mangle* comprises a “pure” species, whereas *R. racemosa* and *R. *×* harrisonii* form a different, nonadmixed species, regardless of the dataset we considered (all samples or only those from the northern coast). The posterior probabilities for trees being classified as F_1_, F_2_ or backcrosses with any parental species were close to zero (Figure 2).

**Figure 3:**
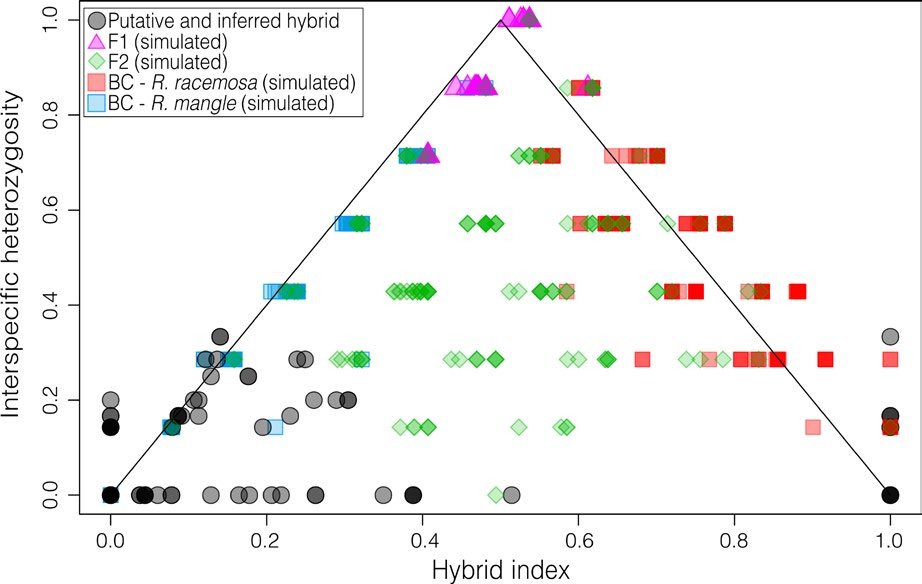
Triangle plot summarizing interspecific heterozygosity against the hybrid index for putative, inferred and simulated *Rhizophora* hybrids. The hybrid index was measured as the proportion of *Rhizophora racemosa* alleles (i.e., 0 indicates individuals with alleles with *R. mangle* ancestry, and 1 denotes *R. racemosa* ancestry). As admixed individuals (gray circles), we considered those individuals from the sympatry zone presenting Q‐values lower than 0.80 for the typical *R. mangle* or *R. racemosa* inferred clusters in the STRUCTURE *K = *2 results and those that were morphologically identified as *R. *× *harrisonii* in the field

DAPC, NEWHYBRIDS and STRUCTURE *K = *3 and *K = *4 results reveal the introgression patterns between the inferred clusters (*R. mangle* on one side and *R. racemosa* and *R. *× *harrisonii* on the other), we estimated hybrid index values for individuals that met our criteria and were identified in the field as *R. *× *harrisonii*. Using a multinomial regression implemented in the INTROGRESS package to estimate individual hybrid indices, we observed a wide range of hybrid index values, that is, ranging from zero to one. Greater variation was observed for *R. mangle* (mean = 0.06235, *SD* = 0.109), whereas all the *R. *×* harrisonii* individuals presented hybrid index = 1 (Figure 3). We plotted hybrid index values against interspecific heterozygosity values obtained from our data and from two‐generation simulated hybrids (F1s, F2s or backcrosses). These simulations showed that when considering only two generations of gene flow between species, there is substantial variation in introgression pattern. When comparing our empirical data with the simulated results, we observed that our experimental results best fit the F_2_ and backcross simulated cases (Figure 3). These results suggest that contemporary hybridization between nonadmixed parental species is uncommon. Additionally, they indicate that F_1_ hybrids are viable and that generally, two or more generations have passed since hybridization started.

Given these partially contrasting results, we performed hierarchical AMOVA to rank models of grouping that could explain the division of independently evolving metapopulation lineages. We grouped individuals based on one a priori hypothesis (*R. mangle, R. racemosa,* and *R. *× *harrisonii* comprise three different groups) and three a posteriori hypotheses (Table 3). When we organized individuals according to their morphological identification carried out in the field (model A), we observed that this grouping explained approximately 66.5% of the total genetic variation. Model B tested the grouping based on DAPC, NEWHYBRIDS, and STRUCTURE *K *=* *4 scenario results (i.e., *R. racemosa* and *R. *× *harrisonii* compose a group separate from *R. mangle*). This model yielded slightly better performance than model A, explaining 67.5% of the total genetic variability. We created models C and D based on model B to contemplate the expected and observed geographic structure of *R. mangle*. We considered previous expectations supported by the genetic subdivision previously described for mangrove species for the same geographic range evaluated herein (Mori, Zucchi, & Souza, 2015; Pil et al., 2011; Takayama et al., *2*013) in model C. This model contrasted w*it*h model D with respect to one *R. mangle* population (from Paracuru) that despite its location within the northeastern coast of southern South America was more genetically similar to samples from the southern populations (see below). Models C and D explained 61.8% and 65.6% of total genetic variation, respectively. Thus, according to our criteria, despite the slight differences among models, the hierarchical AMOVA analysis supports the grouping described by two groups: One composed of individuals identified as *R. mangle* and one composed of individuals with *R. racemosa* and *R. *× *harrisonii* morphological traits (Table 3).

### 3.3 Intragroup genetic structure

**Figure 4:**
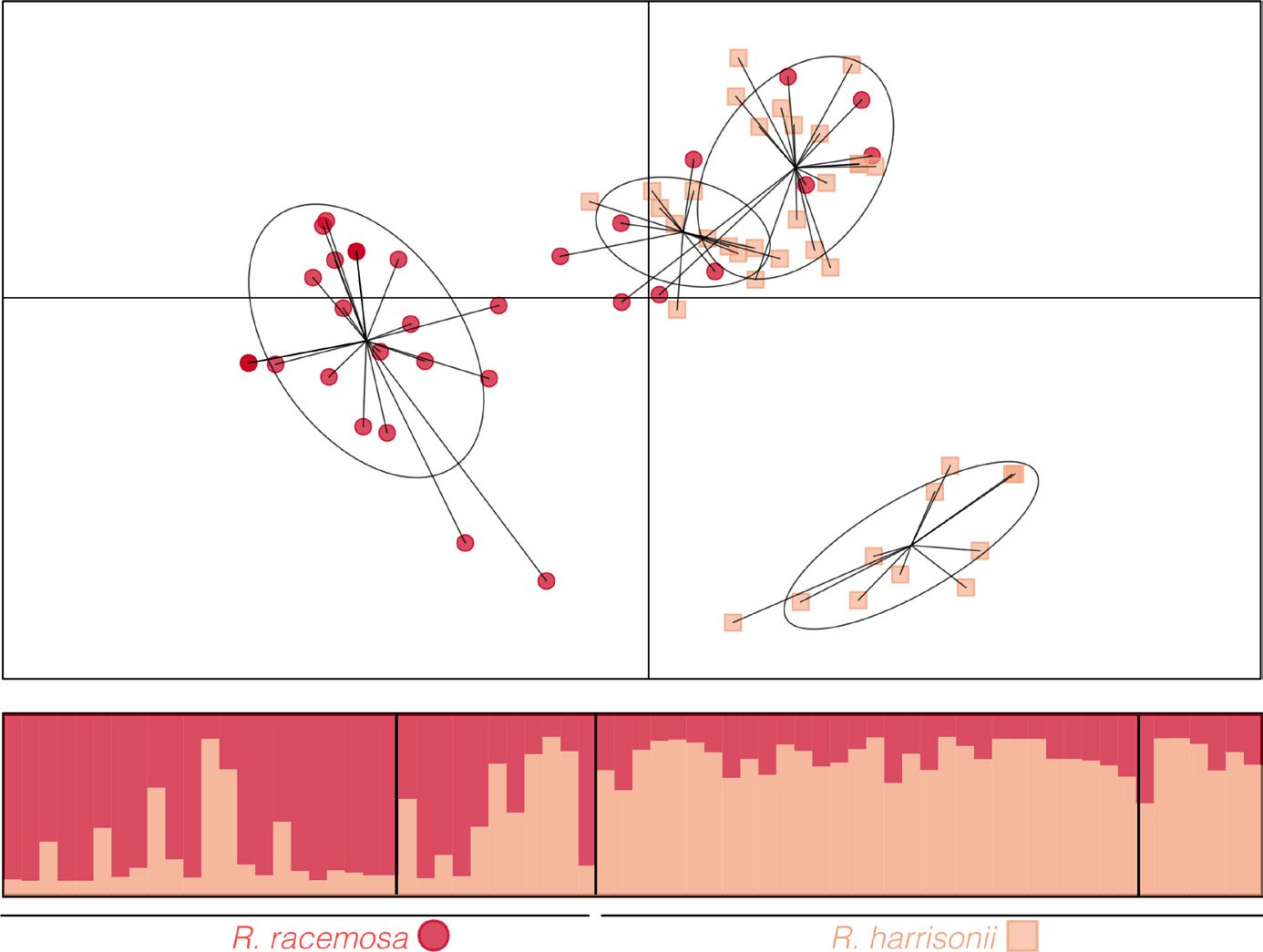
Genetic structure patterns of *Rhizophora racemosa* and *R. *× *harrisonii*. Top: (a) Scatterplot of the discriminant analysis of principal components (DAPC), with the first two principal components represented with red circles and light orange squares representing *R. racemosa* and *R. *× *harrisonii*, respectively. Bottom: Bar plots of STRUCTURE 2.3.4 Bayesian population assignments, with each bar representing a single individual, and each color referring to one inferred group, for two groups (*K *=* *2)

Due to the genetic structure pattern we observed in the three morphologically different *Rhizophora* species from the Neotropics, we conducted intragroup rather than intraspecific analyses. At the intragroup level, there was no evidence of systematic stuttering, null alleles, large allele dropout or LD across samples. For *R. racemosa* and *R. *×* harrisonii*, STRUCTURE and DAPC clustered individuals in groups that largely corresponded to their corresponding morphotypes. The Bayesian clustering method indicated that the *K = *2 scenario better summarized the information than the other *K* scenario (Figure S2), with considerable admixture among inferred groups. Although there was some correspondence between species and assigned group, *R. *×* harrisonii* individuals were more genetically homogeneous, whereas specimens identified as *R. racemosa* presented a wider variation of genetic composition, with some presenting the typical *R. *× *harrisonii* genotype (Figure 4). This admixture pattern was clearer in the DAPC results. According to the multivariate clustering method, a scenario with *K = *4 is a reasonable representation of our data. The inferred population structure showed one cluster that was exclusively composed of *R. racemosa* individuals and a second cluster of *R. *×* harrisonii* plants. The two remaining inferred groups overlapped and both comprise of both morphological species (Figure 4).

The genetic diversities of the *R. racemosa* and *R. *×* harrisonii* populations did not present substantial variation according to the genetic diversity indices (Table 4). In contrast, for *R. mangle,* we observed large differences between samples from the northern and southern coasts of Brazil, similar to results reported previously for this species (Pil et al., 20*11*). Samples from the northern populations exhibited higher genetic diversity summary statistics and a higher frequency of private alleles (Table 4). However, the genetic structure presented some incongruities with previous studies (Mori, Zucchi, & Souza, 2015; Pil et al., 2011; Takayama et al., 2013).

**Table 4:** Population diversity indices

Pop	*N*_e_	*H*_O_	*H*_E_	*F*	Private alleles
RrPNB***	1.562 (0.212)	0.194 (0.053)	0.299 (0.074)	0.229 (0.179)	0
RrMRJ**	2.225 (0.37)	0.349 (0.078)	0.507 (0.066)	0.204 (0.173)	0
RhPNB***	2.534 (0.309)	0.65 (0.148)	0.583 (0.04)	−0.133 (0.279)	0.125
RhMRJ***	2.492 (0.273)	0.509 (0.137)	0.609 (0.05)	0.055 (0.248)	0.25
Total	2.203 (0.157)	0.426 (0.061)	0.5 (0.036)	0.089 (0.11)	
RmPAR***	2.010 (0.26)	0.368 (0.09)	0.436 (0.089)	0.118 (0.101)	0.625
RmALC***	1.638 (0.142)	0.249 (0.051)	0.361 (0.058)	0.312 (0.109)	0.25
RmPNB*	1.741 (0.156)	0.317 (0.082)	0.399 (0.071)	0.184 (0.145)	0
RmPRC***	1.443 (0.165)	0.146 (0.072)	0.249 (0.08)	0.329 (0.167)	0.25
RmTMD	1.000 0	0	0		0
RmVER	1.000 0	0	0		0
RmGPM***	1.085 (0.056)	0.031 (0.016)	0.066 (0.04)	0.191 (0.161)	0.25
RmUBA	1.000 0	0	0		0
RmCNN***	1.032 (0.012)	0.004 (0.004)	0.03 (0.011)	0.872 (0.09)	0
RmPPR	1.000 0	0	0		0
RmFLN	1.011 (0.007)	0.005 (0.005)	0.01 (0.007)	0.489 (0.255)	0
Total	1.269 (0.05)	0.102 (0.02)	0.141 (0.023)	0.311 (0.044)	‐

*N*_e_, number of effective alleles; *H*_O_, observed heterozygosity; *H*_E_, unbiased expected heterozygosity; *F*, fixation index; Private alleles, results for alleles unique to a given population.

The values in the “Total” rows were calculated based on the pooling of the populations of *Rhizophora racemosa* and *R. *× *harrisonii* or the populations of *R. mangle*.

For the Hardy–Weinberg test, *p*‐values are indicated as *p < .05; **p < .01; ***p < .001.

**Figure 5:**
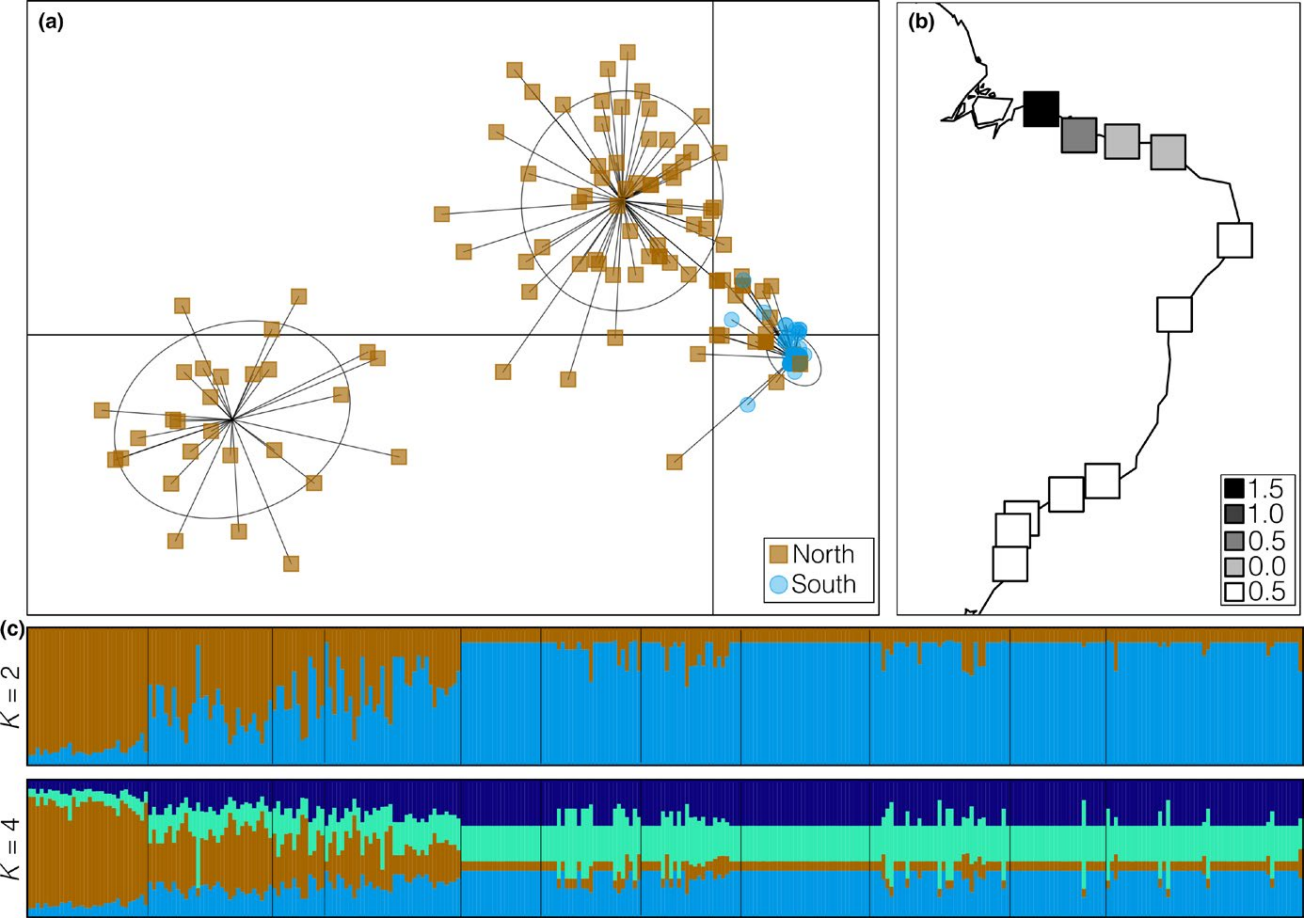
Genetic structure patterns of *Rhizophora mangle*. (a) Scatterplot of the discriminant analysis of principal components (DAPC), with the first two principal components indicated with blue circles, while dark yellow squares represent individuals from populations south and north of the northeastern extremity of South America continent. (b) Summary of the spatial principal component analysis (sPCA) showing the first global principal component, in which squares represent populations, and their colors denote the absolute values of scores. (c) Bar plots of STRUCTURE 2.3.4 Bayesian population assignments, with each bar representing a single individual and each color referring to one inferred group, for two (*K *=* *2) and four (*K = *4) groups

The BIC scores from the DAPC analyses showed that *K = *3 was a reasonable group number for our data. The genetic structure pattern yielded by the multivariate clustering method showed that individuals from the northern populations are more variable than are those from localities south of the NEESA (Figure 5), supporting the genetic diversity indices (Table 4). However, DAPC also clustered together individuals from both regions, indicating that there is no abrupt break between them, as supported by STRUCTURE results. Estimates of lnL and the ad hoc statistic Δ*Κ* presented somewhat discordant but complementary results (Figure S2). Δ*Κ* suggested two groupings that supported our data, *K = *2 and *K = *4, whereas lnL smoothly increased from *K *=* *4 to *K = *7 and then decreased from *K *=* *8 to *K = *10 (Figure S2). As a conservative estimate of population structure, we chose four clusters. Considering both scenarios, *K = *2 and *K = *4, there is a pattern of genetic structure in which populations south of the NEESA are more homogeneous than those from the northern coast (Figure 5). Additionally, the STRUCTURE results showed that within the northern coast, the composition of “southern” gene pools was reduced westward of NEESA.

This westward differentiation was also observed when we explicitly considered the spatial locations of our samples for sPCA. The sPCA results indicated no local structure (*p* = .522) but some global structure (*p* < .001); furthermore, when we plotted the sPCA first positive eigenvalues on a map, the pattern supported the westward gradient, we observed in the northern populations based on the STRUCTURE results (Figure 5). There was an abrupt decrease between the first and second positive eigenvalues, which we interpreted as evidence of strong spatial structure and therefore a reasonable representation of the information we obtained.

## 4 DISCUSSION

Our independent sampling and use of a new set of molecular markers enabled us to build on previous studies on the genetic diversity of *Rhizophora mangle* along the Atlantic coast of South America (Pil et al., 2011; Takayama et al., 2013). The results indicated a genetic subdivision between the populations sampled north and south of the NEESA. This pattern has also been observed in two species of *Avicennia* (Mori, Zucchi, & Souza, 2015)*,* which is a true mangrove genus (Tomlinson, 1986), and *H. pernambucensis* (Takayama et al., 2008), a mangrove associate (Tomlinson, 1986). Our results are consistent with these previous findings, and the present study complements and extends them by analyzing previously unsampled populations along the northern coast and individuals identified as *R. racemosa* and *R. harrisonii* using a newly developed set of microsatellite markers.

### 4.1 *Rhizophora mangle* genetic structure

As expected based on previous findings, we observed that *R. mangle* belongs to two major populations. The subdivision that we observed did not show complete nonoverlap between the populations north and south of the NESSA, with a few admixed individuals found in the northern populations, as observed for *A. germinans* or, more subtly, for *A. schaueriana* (Mori, Zucchi, & Souza, 2015). This trend is evident in the map of the sPCA first eigenvalue scores, which gradually increased westward from the southern population to the northwestern population (Figure 5). Considering the STRUCTURE results, this tendency was also clear for all evolutionary scenarios, that is, considering *K = *2, *K *=* *3, and *K = *4 (Figure 5). Recovering this difference between *Avicennia* (Mori, Zucchi, & Souza, 2015) and *Rhizophora,* genetic structure pattern was possible only because our sampling scheme included two additional localities within the north coast compared with previous studies (Pil et al., 2011; Takayama et al., 2013). Regardless of the method used, genetic structure consisted of an admixture gradient between two gene pools within the northern coast, whereas populations from the southern coast were homogeneous with low genetic diversity (Figure 5 and Table 4).

The major north‐south subdivision observed in the genetic variation of *R. mangle* is likely maintained by superficial ocean currents, as previously suggested for this species (Pil et al., 2011) as well as *A. germinans, A. schaueriana,* (Mori, Zucchi, & Souza, 2015), and *H. pernambucensis* (Takayama et al., 2008) at the same geographic scale. The South Equatorial Current (SEC) branches along the South American coastline at 14–14.5°S, originating the low‐velocity, south‐southwestward Brazil Current (BC), and the high‐velocity, north‐northeastward North Brazil Current (NBC; Lumpkin & Johnson, 2013). As the SEC bifurcates, it facilitates the movement of propagules from southern populations to the north. At the same time, this dynamic imposes a barrier to the westward or southward dispersal of individuals from the northern populations (Mori, Zucchi, & Souza, 2015; Pil et al., 2011). Ocean circulation coupled with different natural history traits may explain the difference in patterns of genetic structure observed between *Avicennia* (Mori, Zucchi, & Souza, 2015) and *R. mangle* (Figure 5)*. Rhizophora* propagules are larger, live longer, survive for at least 1 year in fresh or salt water and require a longer time to become established than *Avicennia* propagules (Rabinowitz, 1978). Consequently, these traits likely allow the elongated, rod‐shaped *Rhizophora* propagules to travel longer distances, increasing the species dispersal potential. This higher dispersal capability could generate the smoother gradient observed along the northern coast of South America, where unidirectional water flow occurs, compared with the almost complete nonoverlap observed for *A. germinans* and *A. schaueriana* (Mori, Zucchi, & Souza, 2015).

The findings presented herein, coupled with previously published research, suggest that oceanic current bifurcation is a key environmental driver that maintains and shapes the genetic diversity of *R. mangle* between the southern and northern populations and within the northern group, corroborating previous studies (Mori, Zucchi, & Souza, 2015; Pil et al., 2011; Takayama et al., 2008, 2013). The processes that originated this pattern of genetic structure, however, are likely to be much more intricate. One possible explanation for the genetic structure and the lower genetic diversity of the southern populations is that northern populations may represent descendants of older populations (refugia) that expanded southwards following postglacial climate changes (Pil et al., 2011). Although our *R. mangle* findings do not reject this process, our analyses considering not only this species but also *R. racemosa* and *R. harrisonii* suggest that an additional process may be influencing *R. mangle* genetic structure. Interspecific hybridization and introgression with *R. racemosa* and *R. harrisonii* may also be a key biological process that influences genetic variation and structure along the Atlantic coast of South America.

### 4.2 *Rhizophora* species complex in Northeastern South America

The genetic differences among *Rhizophora mangle, R. racemosa,* and *R. *× *harrisonii* differences are complex (Cerón‐Souza et al., 2010; Takayama et al., 2013) and much more extensive than those observed in AEP *Avicennia* species (Mori, Zucchi, Sampaio, et al., 2015; Mori, Zucchi, & Souza 2015; Nettel, Dodd, Afzal‐Rafii, & Tovilla‐Hernández, 2008). Although there are morphological (Cerón‐Souza et al., 2010; Tomlinson, 1986) and physiological (Cerón‐Souza et al., 2014) differences between these taxa, species boundaries are permeable (Cerón‐Souza et al., 2010, 2014; Takayama et al., 2013). Our results showed that individuals identified in the field as *R. mangle* comprise a relatively well‐defined genetic cluster that is different from the cluster composed of individuals with *R. racemosa* and *R. *× *harrisonii* morphological traits. This finding became clear when we considered the DAPC clustering and STRUCTURE results according to the lnL criterion, which represented the genetic information of our data in four clusters (*K = *4), and the NEWHYBRIDS classification, wherein *R. mangle* was assigned to one parental species and *R. racemosa* and *R. *× *harrisonii* were assigned to the alternative parental group. Because “hybrids” comprise a heterogenous biological categorization, we further explored introgressive and hybridization processes in this *Rhizophora* species complex by estimating hybrid indexes for admixed individuals and comparing these values with two‐generation simulated hybrids (F1s, F2s or backcrosses). We observed no evidence of first‐generation hybrids between nonadmixed plants, whereas admixed individuals are likely outcomes of second or higher generation hybrids. This result suggests that hybrids are fertile at least toward non‐admixed parental species and that introgression is likely asymmetrical (see below). Moreover, all *R. harrisonii* individuals we studied presented hybrid indexes equal to the nonadmixed *R. racemosa,* which supports DAPC, NEWHYBRIDS, and STRUCTURE (*K = *3 and *K = *4) results from all samples or only considering Northern populations (Figure 2). Additionally, the hierarchical AMOVA results supported this grouping (model B) as the best representation of our data among the a priori and a posteriori hypotheses that we tested. However, this model was only slightly better than model A, which separated *R. mangle, R. racemosa,* and *R. *×* harrisonii,* and model D, which accounted for the previously discussed *R. mangle* geographic subdivision (Table 3).

At a finer scale, regarding the genetic structure within the group composed of *R. racemosa* and *R. *× *harrisonii,* there are two main clusters that correspond to the morphologically identified individuals and two intermediate clusters (Figure 4). This clustering suggests that the traits that are traditionally used to differentiate these morphotypes might be genetically and evolutionarily meaningful. However, due to the porous species boundaries observed in this species complex based on our genetic data and on previous genetic studies (Cerón‐Souza et al., 2010; Takayama et al., 2013), the systematic relationships between these species should be considered with caution.

Under the “unified species concept,” which allows for decoupling of species conceptualization and species delimitation (De Queiroz, 2007), it is difficult to distinguish *Rhizophora* species from the AEP biogeographic region because of their nonreciprocal distinctiveness based on morphological and genetic information. As interspecific hybridization and introgression may interfere with species integrity, and because these processes are pervasive in the studied species complex, we advocate for caution when using these morphological traits for species identification due to the extensive admixture between the *R. racemosa* and *R. *×* harrisonii* groups and considering the entire species complex. Therefore, a synthetic reevaluation of current AEP‐region *Rhizophora* taxonomy would be beneficial, preferably simultaneously considering morphology and genetic data and additional lines of evidence to corroborate or reject the current classification.

The incongruence between species boundaries and morphological traits and its associated taxonomic uncertainty lead to conservation and management issues. In groups without clear delimitations, as it is the case for the AEP *Rhizophora* species complex, it is intrinsically difficult to identify and record individuals using an objective strategy. Thus, we recommend that different scenarios of classification, namely individual morphological “species,” hybrids, groups of species and the entire species complex, should be used as units for applied purposes (Fitzpatrick, Ryan, Johnson, Corush, & Carter, 2015). Regionally, for instance, we believe that using this flexible approach would benefit the Brazilian Mangrove Ecosystem National Action Plan (Plano de Ação Nacional para a Conservação das Espécies Ameaçadas e de Importância Socioeconômica do Ecossistema Manguezal – Brazilian Ministry of the Environment), which is species‐specific and recognizes *R. mangle, R. racemosa,* and *R. *× *harrisonii* as independent entities. Acknowledging that introgression and hybridization are relevant biological processes in this genus would be a useful feature for consideration by conservationists and managers for efficient long‐term conservation plans. Using each grouping may present desirable and unsatisfactory consequences depending on the geographic and temporal scales considered, and thus a case‐by‐case approach is advisable based on conservationists and managers’ objectives.

Our results highlight that hybridization and introgression are major biological processes shaping the genetic diversity of the *Rhizophora* species complex in the Atlantic basin of South America. When we considered the *K = *2 scenario of the STRUCTURE results, admixture was evident between *R. mangle* and the other two species, as was more clearly shown by the INTROGRESS results (Figure 3). This finding suggests that there might be asymmetric introgression toward *R. mangle,* supporting the adaptive evolution hypothesis of this species complex based on physiological traits (Cerón‐Souza et al., 2014). In the Pacific basin, *R. mangle* and F_1_ hybrids presented higher salinity tolerance than did *R. racemosa,* suggesting that introgression and hybridization could be maintained by adaptive evolution (Cerón‐Souza et al., 2014). In the Atlantic basin, *R. racemosa* and *R. *× *harrisonii* are more geographically restricted than *R. mangle,* such that these species are not recorded south to the NEESA, and they are not homogenously distributed in the northern coast of Brazil, where they are found only in estuaries with substantial freshwater input (Menezes et al., 2008). Therefore, our results support the hypothesis that salinity tolerance might be relevant in maintaining this species complex (Cerón‐Souza et al., 2010, 2014).

In addition to blurring species boundaries, a consequence of ancient and ongoing hybridization and introgression could be an increased genetic diversity in populations where the parental species coexist. For example, in East Pacific Central America, ancient interspecific introgression associated with intraspecific population divergence following secondary contact likely increased the genetic diversity of *A. germinans* (Nettel et al., 2008). Likewise, in the *Rhizophora* species complex studied herein, interspecific gene flow, or more specifically, asymmetrical hybridization and introgression toward *R. mangle*, could increase the genetic diversity of the *R. mangle* northern populations. Indeed, we observed higher levels of genetic diversity in these populations (Table 4), supporting independent previous findings (Pil et al., 2011). Thus, although we cannot reject a postglacial southward expansion of mangrove forest distributions in the Atlantic basin of South America as an important evolutionary process shaping the genetic diversity of *R. mangle* (Pil et al., 2011), our results suggest that the evolutionary scenario is more complicated. Our findings highlight that current and historical hybridization and introgression may have increased the level of genetic diversity in northern *R. mangle* populations and altered the distribution of genetic variation between and within populations, possibly creating a bias in *R. mangle* demographic analyses that do not account for these evolutionary processes.

In this study, we recovered and refined *Rhizophora mangle* north‐south genetic structure along the northeastern coast of South America. This subdivision is shared among *R. mangle* (Pil et al., 2011; Takayama et al., 2013), *A. germinans, A. schaueriana* (Mori, Zucchi, & Souza, 2015), and *H. pernambucensis* (Takayama et al., 2008), suggesting that similar extrinsic evolutionary and ecological processes influence how these plants are distributed. Our work highlights the importance of oceanic currents in the dispersal of sea‐dispersed organisms such as *R. mangle*. Finally, we found that past hybridization and introgression processes play important roles in the *Rhizophora* species complex along the northeastern coast of South America. This interspecies gene flow is likely asymmetric, suggesting that adaptation may play a role in maintaining this hybrid zone.

## CONFLICT OF INTEREST

None declared.

## AUTHOR CONTRIBUTION

Conceived and designed the experiments: PMF, GMM,and APS. Performed the experiments: PMF and FMA. Analyzed the data: GMM. Contributed reagents/materials/analysis tools: APS. Wrote the article: GMM. All authors revised, edited and approved the final version of the article.
