# Supplementary figures and images for: Population genetic structure, introgression, and hybridization in the genus Rhizophora along the Brazilian coast
Source: Ecol Evol. 2018 Feb 25;8(6):3491–504. doi: 10.1002/ece3.3900 (PMC5869270; doi:10.1002/ece3.3900)

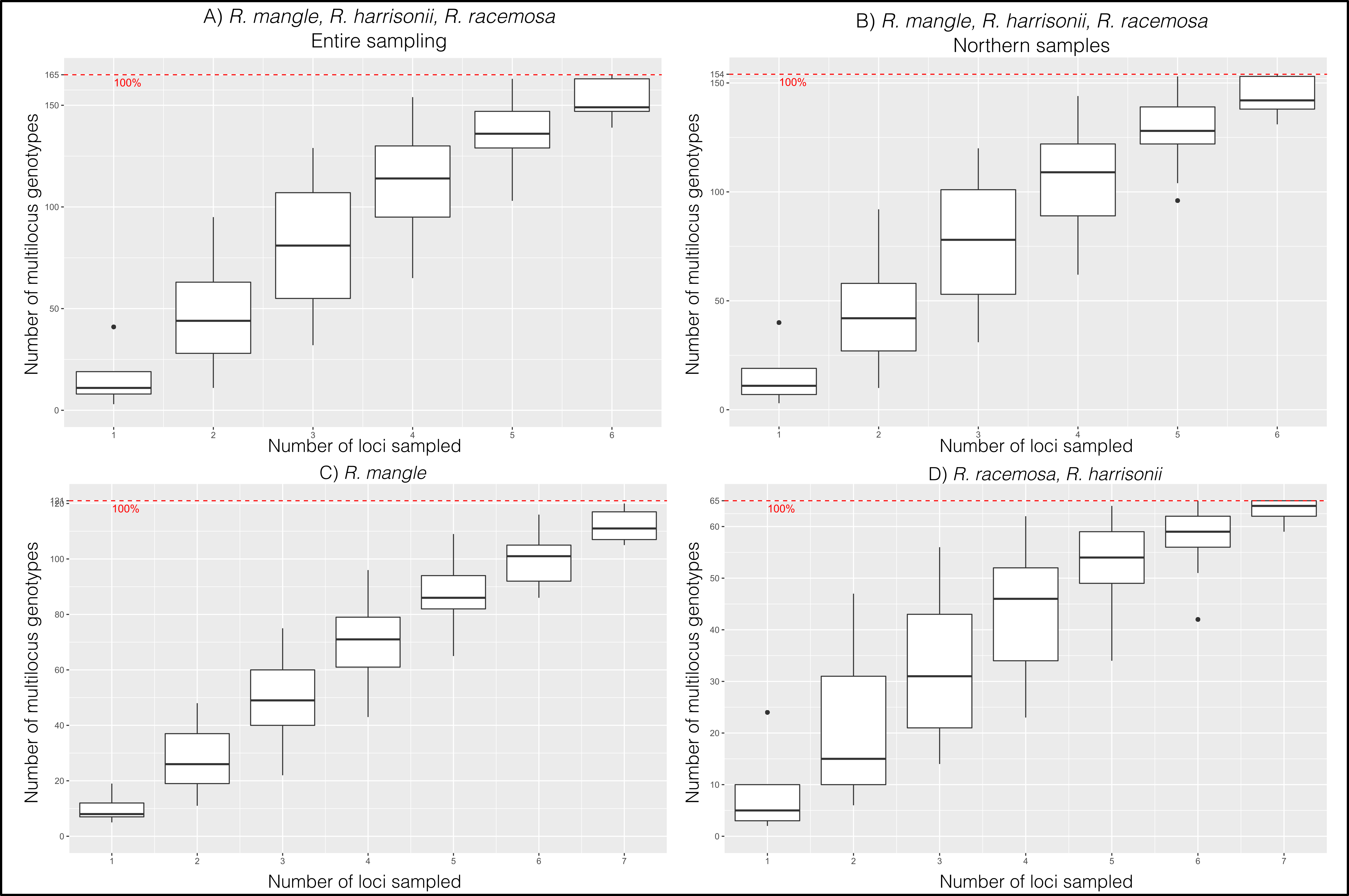

Supplement: Supplementary file 1 [file ECE3-8-3491-s001.png]

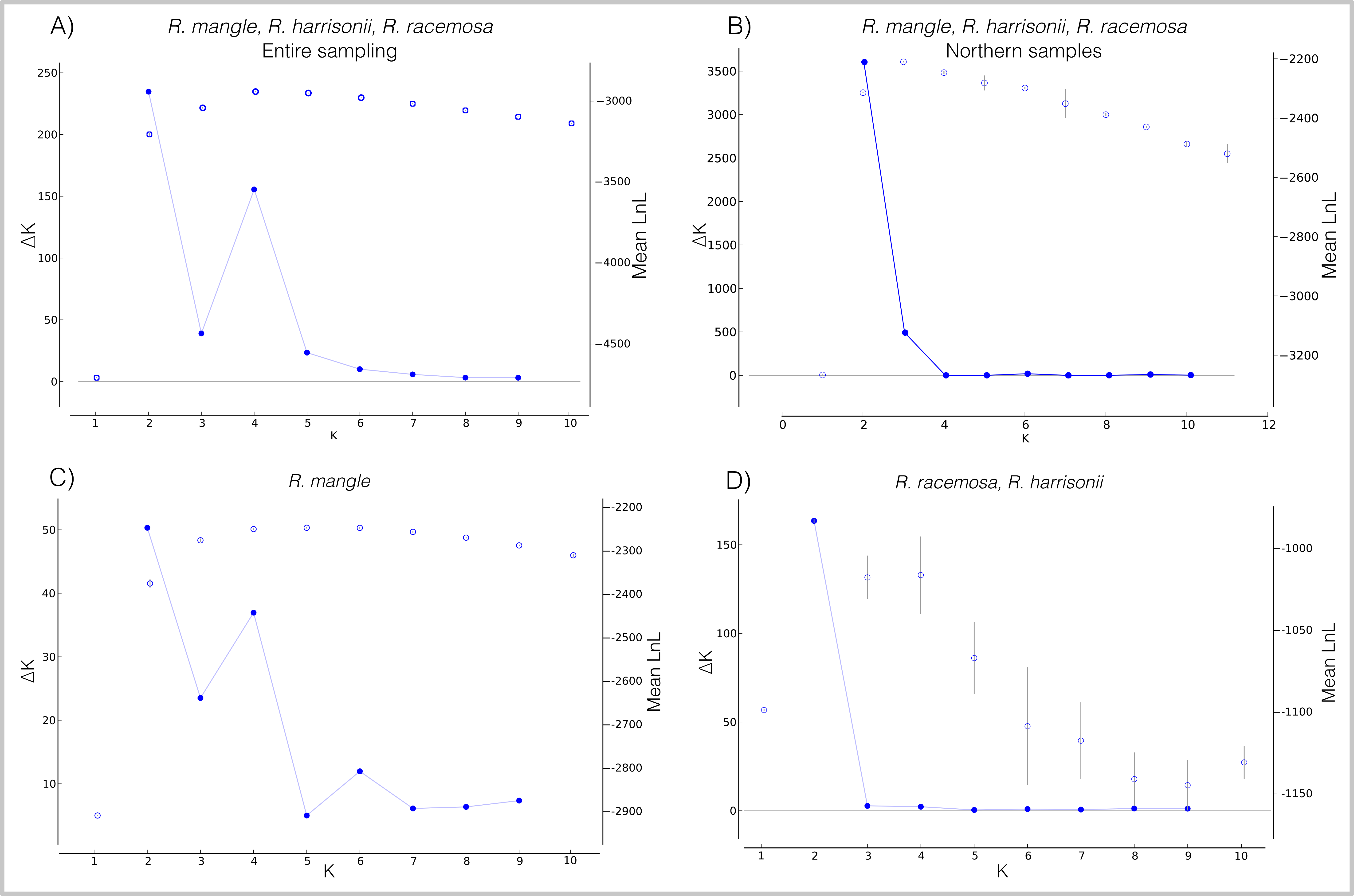

Supplement: Supplementary file 2 [file ECE3-8-3491-s002.png]
