# Supplementary material for: Population genetic structure, introgression, and hybridization in the genus Rhizophora along the Brazilian coast
Source: Ecol Evol. 2018 Feb 25;8(6):3491–504. doi: 10.1002/ece3.3900 (PMC5869270; doi:10.1002/ece3.3900)
Supplement: Supplementary file 3 [file ECE3-8-3491-s003.docx]

**Supporting Information**

**Figure S1.** Genotype accumulation curves for each evaluated dataset. For A) the entire dataset, B) *R. mangle, R. racemosa* and *R.* x *harrisonii* from Northern populations, C) *R. mangle,* and D) *R. racemosa* and *R.* x *harrisonii*, the horizontal axis represents the number of randomly sampled loci, and the vertical axis indicates the number of multilocus genotypes obtained.

**Figure S2.** Bayesian inference of cluster number (*K*) using STRUCTURE. The mean log posterior probability of the data (lnL – individual open circles, right axis) and the ΔK *ad hoc* statistic (interconnected filled circles, right axis) considering A) our entire sample (individuals morphologically identified as *Rhizophora mangle, R. racemosa* and *R.* x *harrisonii*), B) *R. mangle, R.* x *harrisonii* and *R. racemosa* from the northern coast of Brazil, C) only *R. mangle,* and D) only *R. racemosa* and *R. harrisonii*.
